# Alterations in the gut microbiome and metabolism with coronary artery disease severity

**DOI:** 10.1186/s40168-019-0683-9

**Published:** 2019-04-26

**Authors:** Honghong Liu, Xi Chen, Xiaomin Hu, Haitao Niu, Ran Tian, Hui Wang, Haiyu Pang, Lingjuan Jiang, Bintao Qiu, Xiuting Chen, Yang Zhang, Yiyangzi Ma, Si Tang, Hanyu Li, Siqin Feng, Shuyang Zhang, Chenhong Zhang

**Affiliations:** 1 Department of Cardiology, Peking Union Medical College Hospital, Peking Union Medical College & Chinese Academy of Medical Sciences, Beijing, China; 2 Institute of Laboratory Animal Sciences, Chinese Academy of Medical Sciences and Comparative Medicine Center, Peking Union Medical College, Beijing, China; 3 State Key Laboratory of Microbial Metabolism, School of Life Sciences and Biotechnology, Shanghai Jiao Tong University, Shanghai, China; 4 Joint International Research Laboratory of Metabolic & Developmental Science, Shanghai Jiao Tong University, Shanghai, China

**Keywords:** Coronary artery disease, Atherosclerosis, Microbiome, Metabolomics, Diagnostic marker, Multi-omics analysis

## Abstract

**Background:**

Coronary artery disease (CAD) is associated with gut microbiota alterations in different populations. Gut microbe-derived metabolites have been proposed as markers of major adverse cardiac events. However, the relationship between the gut microbiome and the different stages of CAD pathophysiology remains to be established by a systematic study.

**Results:**

Based on multi-omic analyses (sequencing of the V3-V4 regions of the 16S rRNA gene and metabolomics) of 161 CAD patients and 40 healthy controls, we found that the composition of both the gut microbiota and metabolites changed significantly with CAD severity. We identified 29 metabolite modules that were separately classified as being positively or negatively correlated with CAD phenotypes, and the bacterial co-abundance group (CAG) with characteristic changes at different stages of CAD was represented by *Roseburia*, *Klebsiella*, *Clostridium IV* and *Ruminococcaceae*. The result revealed that certain bacteria might affect atherosclerosis by modulating the metabolic pathways of the host, such as taurine, sphingolipid and ceramide, and benzene metabolism. Moreover, a disease classifier based on differential levels of microbes and metabolites was constructed to discriminate cases from controls and was even able to distinguish stable coronary artery disease from acute coronary syndrome accurately.

**Conclusion:**

Overall, the composition and functions of the gut microbial community differed from healthy controls to diverse coronary artery disease subtypes. Our study identified the relationships between the features of the gut microbiota and circulating metabolites, providing a new direction for future studies aiming to understand the host–gut microbiota interplay in atherosclerotic pathogenesis.

**Electronic supplementary material:**

The online version of this article (10.1186/s40168-019-0683-9) contains supplementary material, which is available to authorized users.

## Background

Despite the widespread use of medical therapy in the last decade, cardiovascular diseases (CVDs) remain the leading causes of mortality and morbidity in many developed and developing countries, CVDs remain responsible for 17.7 million deaths every year (constituting 31% of all global deaths), and this number corresponds to one of every three deaths in the US and one of every four deaths in Europe [1]. Coronary artery disease (CAD) refers to the myocardial dysfunction and/or organic lesions caused by coronary artery stenosis and insufficient blood supply. Based on clinical symptoms, the extent of arterial blockage and the degree of myocardial injury, CAD is divided into different categories: stable coronary artery disease (SCAD), unstable angina (UA) and myocardial infarction (MI) [2]. SCAD refers to the syndrome of angina pectoris including recurrent, transient episodes of chest pain reflecting demand-supply mismatch [3]. Patients with spontaneous attacks of prolonged angina-type chest discomfort occurring at rest that are associated with vulnerable plaques are categorized as patients with UA [4]. MI is usually accompanied by severe and persistent chest pain, typical ECG changes, and elevated serum biomarkers of myocardial necrosis like cardiac troponins [5]. UA and MI are also referred to as acute coronary syndrome (ACS) (detailed diagnostic criteria are summarized in Additional file 3). The progression of atherosclerotic plaque is considered to be dynamic and complicated, and the detailed mechanisms underlying the formation, development and dislodgement of plaque are largely unknown. Identifying biomarkers of the risk of plaque destabilization and rupture in patients is important for preventing the transition from coronary stability to instability and the occurrence of thrombosis events.

Recently, multiple studies have suggested that the structure and composition of the gut microbiota in CAD patients exhibit significant alterations. According to a study conducted in Sweden [6], which involved 12 patients and 13 controls, the gut microbiota composition of patients with atherosclerosis (AS) contains relatively high levels of *Collinsella*, whereas that of the normal control group has relatively higher abundance of *Roseburia* and *Eubacterium*. Koren et al. identified *Chryseomonas*, *Veillonella* and *Streptococcus* in AS plaque samples, and several bacterial phylotypes from the gut are common to the atherosclerotic plaque and are correlated with the cholesterol levels [7]. A metagenome-wide association study showed that the abundance of *Enterobacteriaceae* and *Streptococcus* spp. were higher in patients with atherosclerotic cardiovascular disease than in healthy controls [8]. We hypothesize that the reason why discrepancies on microbial signatures of different atherosclerotic populations are due to the intrinsic flaw of taxon-based analysis, which overlooks the variations of the bacterial strains belonging to the same taxon. Moreover, the resident microbial communities in the intestinal tract act as key “metabolic filters” of the diet as these species can convert common nutrients to metabolites, and specific microbial-associated metabolites, such as trimethylamine-N-oxide (TMAO), short-chain fatty acids (SCFAs) and secondary bile acids, have been shown to affect the progression of CVD [9–13]. For example, TMAO, an independent marker for predicting clinical vascular events, has been mechanistically linked with the development of atherosclerosis in humans and mice. This substance is generated when a toxic metabolite (trimethylamine) produced by bacterial fermentation of dietary fat-derived choline enters the host bloodstream and is metabolized by the liver [14]. Both epidemiological and animal studies have provided strong evidence showing that alterations of the gut microbiota might be involved in the development of atherosclerosis, but the features of the gut microbiota in patients with different categories of CAD remain to be determined.

To address the questions above, we analysed the gut microbial characteristics of 161 CAD patients (SCAD group *N* = 44, UA group *N* = 80, and MI group *N* = 37) and 40 healthy controls through high-throughput sequencing. In addition, we used untargeted liquid chromatography-mass spectrometry (LC-MS) to analyse the metabolic profiles of these patients. Based on these multi-omic analyses, we identified specific features of the gut microbiota and host metabolite profiles that are associated with increases in CAD severity and further established relationships, particularly between several bacterial co-abundance groups (CAGs) and serum metabolite function modules. This information may be used to construct a disease classifier for discriminating between healthy controls and different CAD subgroups (an overview of the workflow is provided in Additional file 1: Figure S1). Our study reveals that the integration of metabolomic and 16S rRNA V3-V4 sequencing analyses might reveal the interactions that occur between the host and the gut microbiome.

## Results

### Characteristics of the study population

A total of 201 participants were enrolled at Peking Union Medical College Hospital and were further divided into the following four groups based on guidelines for diagnosis (detailed in the “Materials and methods” section): control group (*N* = 40), SCAD group (*N* = 44), UA group (*N* = 80), and MI group (*N* = 37). The traditional cardiovascular risk factors of the 201 subjects are summarized in Table 1, and the extrinsic host factor profiles, including diet, lifestyle, and stool consistency, are summarized in Additional file 2: Table S1. Compared with the healthy subjects, the patients in the SCAD, UA and MI groups showed disruptions in glucose and lipid metabolism and an increased inflammatory state. Except for the significant differences in the hs-CRP levels between SCAD vs. MI and UA vs. MI, the risk factors showed no significant difference between comparisons of CAD subgroups. The atherosclerosis burden was quantified using the Gensini score [15], and the median scores of the various groups were as follows: SCAD, 35.25 (24, 65.5); UA, 44.25 (33, 60); and MI, 62.5 (47, 74.5). We observed that the Gensini score level increased significantly with the development of atherosclerosis and showed significant difference between SCAD vs. MI (*P* < 0.001, Mann-Whitney *U* test) and UA vs. MI (*P* <0.05, Mann-Whitney *U* test) (Additional file 1: Figure S2). We also found that the MI group exhibited a high proportion of three-stenosed vessels (51.4%), which was consistent with the coronary atherosclerotic burden observed in other populations diagnosed with CAD [16]. Cardiac troponin I (cTnI) has been found to have excellent sensitivity and specificity as an indicator of myocardial necrosis [17], the median levels of cTnI in our study were 0, 0.005 (0, 0.02), 0.003 (0, 0.014) and 0.08 (0.06, 1.1) μg/L from Control subjects, SCAD, UA to MI patients, respectively. And significant differences in the cTnI levels were found in all pairwise comparisons with the exception of the SCAD vs. UA. (SCAD vs. MI, *P* < 0.001; UA vs. MI, *P* < 0.001; Mann-Whitney *U* test). According to the results of cardiac catheterization and biochemical data, we suppose that the integration of the Gensini score, number of stenosed vessels and cTnI level can indicate the severity of CAD.

**Table 1:** Characteristics of the study cohort

	Control (*n* = 40)	SCAD (*n* = 44)	UA (*n* = 80)	MI (*n* = 37)	*P* value for trend
Age, years^†^	55 (49, 62.25)	62.5 (52.5,68.8)	62.5 (57.3, 67.8)	63 (53.5, 72)	0.023^b^
Female^§^	23 (57.5)	11 (25)	24 (30)	8 (21.6)	0.002^abc^
SBP, mmHg^*^	119.9 ± 10.8	130.8 ± 15.5	131.1 ± 17.7	126.5 ± 16.5	0.002^abc^
BMI, kg/m^2*^	24.2 ± 2.9	25.1 ± 3.3	26.7 ± 2.9	26.1 ± 3.8	< 0.001^bc^
Waistline, cm^*^	83.3 ± 10.2	90.1 ± 7.8	93.9 ± 8.7	93.6 ± 9.7	< 0.001^abc^
Current smoker^§^	6 (15)	25 (56.8)	43 (53.8)	22 (59.5)	< 0.001^abc^
Drinking history^§^	6 (15)	21 (47.7)	38 (47.5)	22 (59.5)	< 0.001^abc^
No. of stenosed vessels^§^					0.126
NA	NA	3 (6.8)	5 (6.3)	0 (0)	
1	NA	13 (29.5)	26 (32.5)	5 (13.5)	
2	NA	11 (25)	15 (18.8)	13 (35.1)	
3	NA	17 (38.6)	34 (42.5)	19 (51.4)	
Gensini score^†^	NA	35.25 (24, 65.5)	44.25 (33, 60)	62.5 (47, 74.5)	< 0.001^de^
Medication					
Statins^§^	2 (5)	13 (29.5)	28 (35)	11 (29.7)	0.005^abc^
Antihypertensive drugs^§^	8 (20)	28 (63.6)	49 (61.3)	23 (62.2)	< 0.001^abc^
Oral antidiabetic drugs^§^	2 (5)	12 (27.3)	15 (18.8)	12 (32.4)	0.014
Laboratory data					
TG, mmol/l^†^	1.3 (0.86, 1.87)	1.25 (1, 1.6)	1.6 (1.1, 1.9)	1.3 (1.1, 2.1)	0.113
TC, mmol/l^†^	4.7 (4, 5.3)	3.7 (3.2, 4.6)	3.8 (3.3, 4.5)	4 (3.3, 4.7)	0.001^abc^
HDL-C, mmol/l^†^	1.1 (0.9, 1.4)	1 (0.8, 1.2)	0.9 (0.8, 1.1)	0.9 (0.8, 1.1)	< 0.001^abc^
LDL-C, mmol/l^†^	2.8 (2.2, 3.2)	2.1 (1.7, 2.7)	2.2 (1.7, 2.7)	2.3 (1.6, 2.8)	0.013^b^
FBG, mmol/l^†^	6.2 (5.3, 7.9)	7.05 (5.9, 8.4)	6.4 (5.4, 7.9)	7.9 (6.2, 10.2)	0.019 ^c^
BUN, mmol/l^†^	4.9 (4.3, 5.9)	5.9 (4.9, 6.8)	6.2 (4.9, 7.3)	5.7 (5, 7)	0.006^bc^
CR, μmol/l^†^	68.5 (61.2,79.8)	78.5 (67.3,92.8)	81.5 (68.25, 90)	79 (70.5, 89.5)	0.01^bc^
cTnI, μg/l^†^	0	0.005 (0, 0.02)	0.003 (0, 0.014)	0.08 (0.06, 1.1)	< 0.001^abcde^
hs-CRP, mg/l^†^	0.7 (0.4, 1.2)	1.3 (0.6, 3.2)	1.9 (0.8, 2.9)	3.8 (2, 19.4)	< 0.001^bcde^
TNF-α, pg/mL^†^	11.4 (3.1, 21.9)	25.9(15.2, 64.2)	22.6 (15.8, 38.9)	18.8 (14.3, 23.4)	< 0.001^abc^

^†^median (IQR), ^*^mean ± SD, ^§^*n* (%)

Continuous, normally distributed variables among the four groups were analysed by a one-way analysis of variance. The Kruskal-Wallis H-test was applied for data of this type that were not normally distributed. Continuous, normally distributed variables between two groups were analysed by Student’s t-test. The Mann-Whitney *U* test was applied for data of this type that were not normally distributed. Categorical variables were compared by the *χ*^2^ test. *N*/*A* not available. Drinking history is defined as patients who consumed ≥ 50 g of alcohol per day. ^a^*P* < 0.05 for equality between SCAD vs. control. ^b^*P* < 0.05 for equality between UA vs. control. ^c^*P* < 0.05 for equality between MI vs. control. ^d^*P* < 0.05 for equality between SCAD vs. MI. ^e^*P* < 0.05 for equality between UA vs. MI.

### Changes in the serum metabolomic features between CAD subgroups

To identify the serum metabolome features of the patients in different CAD categories, untargeted metabolome profiles were generated on fasting serum samples by LC-MS. Considering the variable stability of metabolites and in order to collect all possible metabolites in serum, we optimized the sample preparation and detection for both polar ionic and lipid modes. Metabolomic (polar ionic mode) and lipidomic (lipid mode) profiling yielded 7061 features and 4975 features, respectively. We conducted a “cross-comparison scheme”, in which the various stages of CAD were compared with normal coronary arteries and to each other: control vs. SCAD for plaque formation and growth, SCAD vs. UA for transition from coronary stability to instability, SCAD vs. ACS for plaque rupture and erosion, and UA vs. MI for cardiac events [18]. Based on the OPLS-DA models of metabolite profiling data, we found that the serum metabolites were significantly different between all patients with CAD and healthy controls. The patients with SCAD status exhibited significantly different metabolite profiles compared with the healthy subjects and the patients with ACS. Moreover, the patients with UA and MI, which are two different stages of ACS, also showed significant differences (Additional file 1: Figures S3 and S4).

**Fig. 1:**
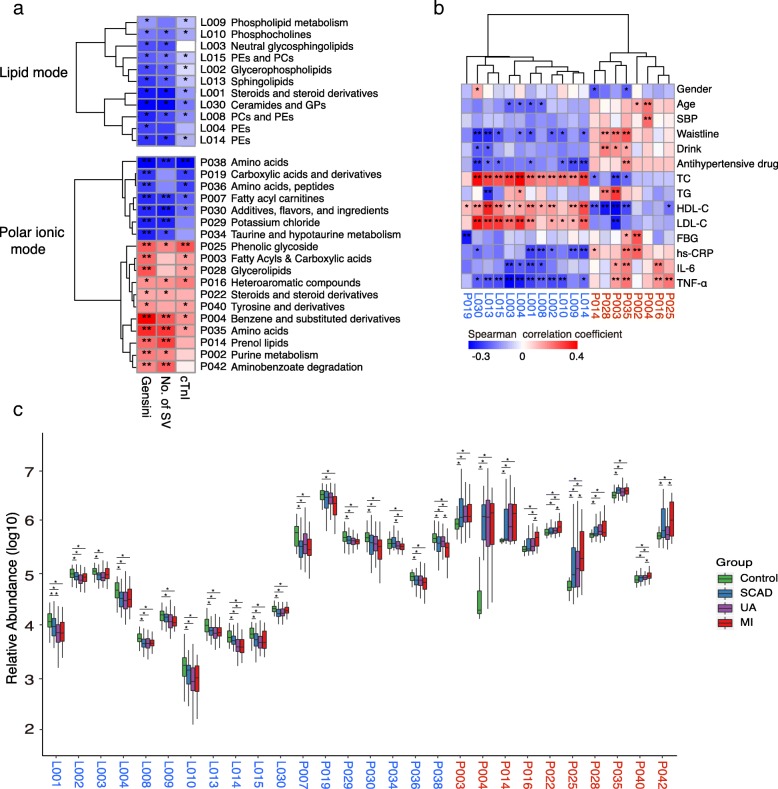
Identification of the major serum metabolite modules associated with the onset and development of CAD. **a** Spearman correlations between serum metabolite modules and major CAD phenotypes. **b** Spearman correlations between serum metabolite modules and major CAD risk factor indicators. **c** The box plot shows that the serum metabolite modules significantly changed between different groups according to the Wilcoxon rank sum test. The names of the metabolite clusters comprising the CAD-positive and CAD-negative metabotypes are highlighted in red and blue, respectively. In **a** and **b**, the colour represents positive (red) or negative (blue) correlations, and FDRs are denoted as follows: *FDR < 0.05, **FDR < 0.01. In **c**, the asterisk represents *P* values < 0.05 by the Wilcoxon rank sum test, boxes represent the inter-quartile ranges, and lines inside the boxes denote medians. PE phosphatidylethanolamine, PC phosphatidylcholine, GP glycerophospholipids, SBP systolic blood pressure, TC total cholesterol, TG triglyceride, HDL-C high-density lipoprotein cholesterol, LDL-C low-density lipoprotein cholesterol, FBG fasting blood glucose, hs-CRP high-sensitivity C-reactive protein, IL-6 interleukin 6, TNF-α tumour necrosis factor-α

From the OPLS-DA models, we identified two collections of differentially produced compounds that included 334 metabolites (122 known and 212 unknown) under polar ionic mode and 494 metabolites (111 known and 383 unknown) under lipid mode. The metabolic features identified in the analysis included both host-derived and bacterial-derived metabolites. We binned these serum metabolites into 72 co-abundance clusters across all the subjects. We identified 29 of the 72 metabolite clusters (40.3%) to be significantly associated with the Gensini score, number of stenosed vessels and cTnI levels (Fig. 1a, Additional file 2: Tables S2 and S3). Among these 29 clusters, the metabolite clusters under polar ionic mode were separated into two groups that were either positively (CAD enriched) or negatively (control enriched) correlated with CAD severity, while the metabolite clusters under lipid mode among these 29 clusters were only negatively correlated with CAD severity (Additional file 2: Table S4). Moreover, the CAD-enriched metabotypes were positively correlated with the main risk factors of CAD but negatively correlated with cholesterol. For example, the metabolite module P003 (fatty acyls and carboxylic acids) was positively correlated with the waistline (Rho = 0.29, adjusted *P* value < 0.001), triglyceride (TG) (Rho = 0.4, adjusted *P* value < 0.001) and TNF-α (Rho = 0.22, adjusted *P* value = 0.009) but negatively correlated with HDL-C (Rho = − 0.38, adjusted *P* value < 0.001). While the control-enriched metabotypes generally showed the opposite correlation (Fig. 1b, Additional file 1: Figure S5).

By abundance comparison, we found that all the CAD-negative metabotype modules were generally highly abundant in the healthy subjects. Among the CAD-positive-associated metabotypes, for Control vs. SCAD, the metabolism changed for fatty acyls and carboxylic acids, benzene and substituted derivatives, prenol lipids, phenolic glycoside, and amino acids, including L-leucine and aminobenzoate degradation; the comparison of SCAD vs. UA did not identify much modules with significant changes; for UA vs. MI, heteroaromatic compounds, steroids, phenolic glycoside, tyrosine and derivatives, and aminobenzoate degradation modules were elevated (Fig. 1c).

Taken together, the results suggested that the CAD patients had significantly different metabolite profiles compared with healthy controls, and the metabolite levels may further shift with different CAD severity.

### Changes in the gut microbiome between the CAD subgroups

**Fig. 2:**
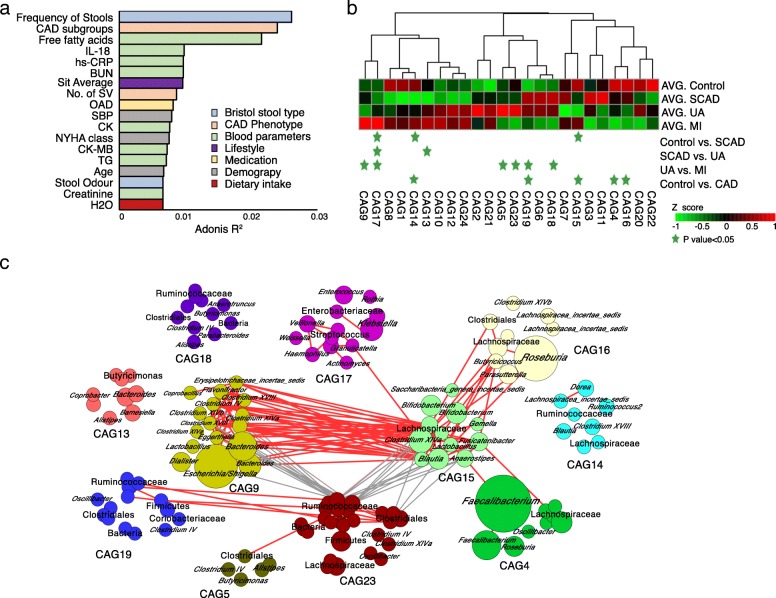
Identification of the important co-abundance groups that were strikingly different across CAD groups. **a** Bar plot illustrating the top host factors that were found to be significantly associated with gut microbial variations. The variations were derived from between-sample unweighted UniFrac distances. The bars were coloured according to metadata categories. Size effects and statistical significance were calculated by PERMANOVA (Adonis). The *P* value was controlled at 0.1. **b** Relative abundances of the 24 co-abundance groups (CAGs) across different CAD subgroups. The abundance profiles were transformed into Z scores by subtracting the average abundances and dividing the standard deviations of all the samples. The *Z* score was negative (shown in green) when the row abundance was lower than the mean. CAGs at *P* values <0.05, as determined by the Wilcoxon rank sum test, are marked with green stars. **c** OTU-level network diagram showing the enrichments of OTUs in the different groups based on significantly changed CAGs. Node size indicates the mean abundance of each OTU. The bacteria denoted on the nodes were of the lowest classification status that could be clearly identified using the RDP classifier. Lines between nodes represent correlations between the nodes connected by the lines, with line width indicating correlation magnitude, red representing positive correlation, and grey representing negative correlation. Only lines corresponding to correlations with magnitudes greater than 0.4 were drawn. IL-18 interleukin 18, BUN blood urea nitrogen, hs-CRP high-sensitivity C-reactive protein, OAD Oral antidiabetic drugs, SBP systolic blood pressure, CK creatine kinase, NYHA class New York Heart Association classification, TG triglyceride

As shown in the results, many CAD-associated metabotypes are involved in the metabolism of aromatic compounds, which may be co-metabolites of the gut microbiota and the host. We then investigated the changes in the gut microbiome in the CAD subgroups by sequencing the faecal 16S rRNA V3-V4 region. No significant differences in the richness and diversity of the gut microbiota were found between the healthy control subjects and the patients with SCAD, while the UA group exhibited higher values of observed operational taxonomic units (OTUs) and a higher Chao1 index than the healthy control group (Additional file 1: Figure S6). To assess the overall structure of the gut microbiota, the score plot of the principal coordinate analysis based on unweighted UniFrac distances was constructed, and the results showed that with intensification of the pathophysiological process of coronary AS, the structure and composition of the microbiota differed significantly (Additional file 1: Figure S7). We explored the associations between variations in the gut microbiota and host characteristics using Adonis. Eighteen parameters were significantly associated with gut microbial variations derived from between-sample unweighted UniFrac distances (*P* < 0.1 of PERMANOVA, Fig. 2a, Additional file 2: Table S5). Bristol stool type, CAD phenotype indicators, inflammatory factors, lifestyle and medication use were among the strongest explanatory factors, which was consistent with the results observed for Western populations [19].

As bacteria work as functional groups (guilds) in the gut ecosystem [20], we next constructed a co-abundance network in which the 274 OTUs were shared by at least 20% of the samples based on SparCC correlation coefficients and clustered the OTUs into 24 CAGs. Of these, CAG4, CAG14, CAG15 and CAG16 decreased significantly in patients with CAD compared with the healthy controls (Wilcoxon rank sum test, *P* < 0.05, Fig. 2b). Of the OTUs in these CAGs, 81.6% belonged to Lachnospiraceae and Ruminococcaceae (Fig. 2c), members of which may protect against inflammation by producing butyric acid [21, 22]. Then, we analysed CAGs with significant abundance differences in different subgroups (Fig. 2b). Notably, the abundance of CAG17 was significantly higher in the group with more severe disease. This CAG comprised many Proteobacteria phylotypes (Fig. 2c), such as *Klebsiella*, *Streptococcus*, *Haemophilus* and *Granulicatella*, members of which have been reported as pathogens or opportunistic pathogens [23–26]. Through Spearman correlation analysis, we did not identify any CAGs that were directly correlated with the three major phenotype indicators of CAD. However, we showed that the CAGs had significant correlation with age, inflammatory markers (hs-CRP and IL-18), blood lipids and dietary fibre intake (Additional file 1: Figure S8).

### Multi-omic network analysis reveals the relationship between the gut microbiota and serum metabolites in CAD

**Fig. 3:**
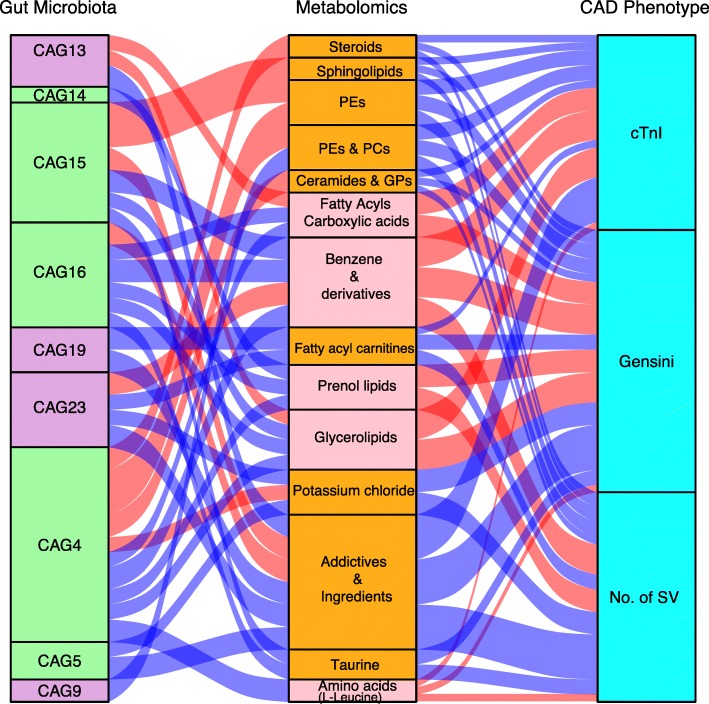
Interrelationship between gut microbiota composition, host metabolic profile and main CAD phenotype. Visualization of the correlation network according to Spearman correlation analysis between the gut microbiota of significant CAGs and the parameters represented CAD severity was mediated by serum metabolites. Red connections indicate a positive correlation (Spearman correlation test, FDR < 0.05), while blue connections show correlations that were negative (Spearman correlation test, FDR < 0.05). In the gut microbiota column, the green stratum represents CAGs that were highly enriched in the control group, and the stratum coloured in purple was increased in the more severe group among the subgroup’s comparisons. In the metabolomics column, the orange stratum represents CAD-negative metabotypes, and the pink stratum represents CAD-positive metabotypes. CAG co-abundance group, PE phosphatidylethanolamine, PC phosphatidylcholine, GP glycerophospholipids, No. of SV number of stenosed vessels, cTnI troponin I

We subsequently assessed the correlation between the gut microbiota and serum metabolites to further explore the characteristics of the microbiota in patients with different CAD severities. Given an FDR of 5%, 9 gut microbiota CAGs were significantly correlated with 14 metabolic modules, as demonstrated through Spearman correlation coefficients, and these metabolic modules were further correlated with the Gensini score, number of stenosed vessels or cTnI level, which can represent the CAD severity (Fig. 3 and Additional file 2: Table S6).

CAG4, CAG14, CAG15 and CAG16, enriched in the control group, were positively correlated with metabotypes that were “CAD-negative associated”, such as sphingolipids and PEs, but negatively correlated with “CAD-positive-associated metabotypes”, such as glycerolipids, prenol lipids and benzene derivatives. In particular, CAG4, mainly composed of *Faecalibacterium* and *Roseburia*, was closely related to 10 serum modules, which implies that CAG4 might play an important role in the maintenance of the normal coronary artery physiological conditions by interacting with different serum metabolites.

The analysis of the CAGs that were increased in the more severe groups showed that these were negatively correlated with the module composed of additive flavours and ingredients, including linalyl cinnamate and gingerol. Recent studies have demonstrated that these food flavourings undergo transformation in the gut microbiota and thereby acquire additional properties that promote the biological activities of these compounds [27, 28]. For instance, CAG9, composed of several genera belonging to Clostridium, was negatively correlated with glycerophospholipids such as PE (22:0/14:0) and PC(P-16:0/20:2). CAG13, represented by *Butyricimonas*, was found to be positively associated with carboxylic acids, steroids and glycerolipid metabolites such as Ne, Ne dimethyllysine, glycerol 1-hexadecanoate and 1b-hydroxycholic acid. CAG19 and CAG23 were both negatively correlated with fatty acyl carnitines, mainly L-octanylcarnitine, and CAG23 was also positively correlated with benzene and substituted derivatives.

As mentioned previously, the gut bacterial CAGs were not directly correlated with the three major phenotype indicators of CAD. The concerted changes within the microbiome and metabolome allowed us to construct interaction networks for CAGs and the CAD-associated metabolite modules, indicating that the gut microbiota may influence CAD severity by interacting with host metabolites.

### Subgroup identification and prediction based on CAGs and CAD-associated metabotypes

To determine whether the gut bacterial CAGs and metabolite modules can be regarded as identification biomarkers for distinguishing various stages of CAD from normal coronary arteries and from each other, random forest models were constructed to classify different stages of CAD based on 24 CAGs and 72 serum metabotypes, and receiver operating characteristic (ROC) curves were used to test the classification (details are shown in the “Materials and methods” section). We mainly established five models, namely, Control vs. CAD, Control vs. SCAD, SCAD vs. UA, SCAD vs. ACS and UA vs. MI.

**Fig. 4:**
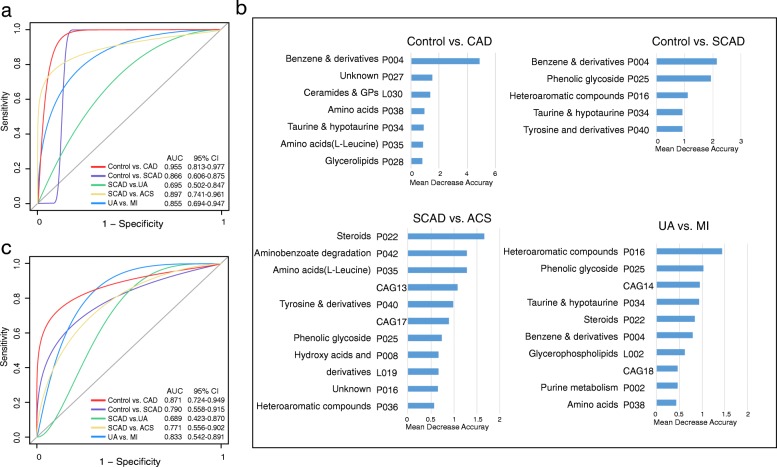
Diagnostic outcomes are shown via receiver operating characteristic (ROC) curves for CAD severity. **a** ROC of the random forest classifier using CAG + serum metabolite modules based on the most important variables by ranking the variables by importance in the discovery phase among 201 subjects. **b** The detailed explanatory variables based on the random forest model in each comparison. The lengths of the bars in the histogram represent the mean decrease accuracy, which indicates the importance of the CAG or metabolite module for classification. **c** ROC of the cross-validated random forest classifier using the most important explanatory variables in the validation cohort. GP glycerophospholipids

We could accurately distinguish CAD patients from healthy controls, as indicated by the area under the receiver operating curve (AUC), which had a value up to 0.955 (Fig. 4a). Among the strongest discriminatory features, benzene and substituted derivatives had the greatest impact, followed by metabotypes such as ceramides, glycerophospholipids, taurine and amino acids, including L-leucine and L-proline. (Fig. 4b). In the subgroup comparisons, we considered control vs. SCAD for plaque formation and found that SCAD patients possessed distinct features compared with the controls (Fig. 4a). The features with predictive value were metabolic modules, including benzene and substituted derivatives, phenolic glycoside, heteroaromatic compounds, taurine and tyrosine (Fig. 4b). Then, we focused on SCAD vs. ACS for the transition from coronary stability to instability, and the AUC for this comparison was 0.897 (Fig. 4a). The main features included steroids, aminobenzoate degradation, amino acids (L-leucine, L-proline and glutamylserine), tyrosine and derivatives, CAG17 and CAG13 (Fig. 4b). The AUC for the classification of MI from the UA was 0.855 (Fig. 4a), and in predicting the process for cardiac events, metabolite modules were mainly annotated to heteroaromatic compounds, phenolic glycoside, taurine, steroids, CAG14 and CAG18 (Fig. 4b). However, we obtained poor performance when discriminating between SCAD and UA due to decreased specificity and sensitivity (Fig. 4a). Notably, we found that these markers were common microbial and metabolic characteristics of CAD subgroups and contributed greatly to the identification of plaque formation and rupture even with myocardial ischaemia.

Subsequently, we enrolled another independent validation cohort that met the same inclusion and exclusion criteria as the discovery phase (Additional file 2: Table S7). The validation cohort was also divided into the control group (*N* = 12), SCAD group (*N* = 11), UA group (*N* = 11) and MI group (*N* = 3). We used the established random forest models to further demonstrate the potential ability of subgroup identification. Consistently, the features of the CAG + metabolite module can help distinguish CAD patients vs. healthy controls, SCAD vs. control, ACS vs. SCAD and MI vs. UA (Fig. 4c). Similarly, the performance on SCAD and UA individuals was not as satisfactory.

Overall, the CAD-associated microbial and metabolic features captured by the classifier offered further evidence of the dysbiotic gut microbiome and highlighted its great potential for the detection of various stages of CAD.

## Discussion

In the current study, we demonstrated that CAD patients had significantly different serum metabolite profiles and gut microbiota compared with healthy controls and showed that the metabolites and gut microbiota may further shift during the development of CAD. Through multi-omics analyses, our study found that CAGs and metabotypes that exhibited significant changes with the development of CAD were significantly correlated and might be used independently as biomarkers for CAD subtype diagnosis.

We confirmed that the structural characteristics of the gut microbiota were altered with the development of CAD compared with those of healthy controls. The abundance of CAG17 increased with CAD severity. This CAG contained several gram-negative bacteria, such as *Veillonella*, *Haemophilus* and *Klebsiella* and these bacteria trigger the innate immune response via lipopolysaccharide (LPS) production and elicit a subsequent inflammatory reaction that is mediated by local generation of cytokines [29]. *Klebsiella* is also reported to be associated with disease in hypertensive populations and is responsible for hypertension pathology [23]. Notably, we did not find any significant correlation between CAG17 and CAD-associated metabolic models, which suggested that these bacteria might contribute to CAD development by inducing endotoxaemia and systemic inflammation [30–32]. Our data also showed that 4 CAGs containing OTUs from Lachnospiraceae and Ruminococcaceae, which are major members of the human GI tract that produce butyric acid [33], were significantly reduced with CAD development. A recent study involving the TwinsUK cohort revealed that OTUs belonging to the Ruminococcaceae family are negatively associated with pulse wave velocity (PWV), which is a measure of arterial stiffness [34]. Among the bacteria in these CAGs, *Roseburia* has been associated with weight loss and reduced glucose intolerance in mice, and a strong anti-inflammatory effect of *Faecalibacterium prausnitzii* has been demonstrated both in vitro and in vivo [35]. Interestingly, another study showed that the abundances of *Clostridium IV*, *Clostridium XlVa* and *Clostridium XVIII*, which also belong to Ruminococcaceae, were higher in patients with coronary heart disease [36]. In the current work, we also found that CAG9, CAG19 and CAG23, which were also composed of OTUs from Ruminococcaceae, were enriched significantly in patients with severe disease. In fact, even though the OTUs were assigned to the same genus, their functions may be distinct because the functions of bacteria are strain specific [37]. Ecologically, gut bacteria do not exist in isolation but rather as functional groups named “guilds”. The key members of a co-abundance group would thrive or decline together in response to the changing physiological environmental resources and form different guilds [38]. Therefore, compared with the conventional taxon-based analysis, the CAG-based analysis performed in this study offers a more ecologically relevant method for reducing the dimensionality of microbiome datasets and facilitate the identification of functionally important members of the gut microbiota in CVD. In summary, our data suggested that the composition of the gut microbiome also changes dynamically with chronic development of CAD.

The human gut microbiota interacts extensively with the host through metabolic exchange and substrate co-metabolism. The human metabolome is composed of endogenous metabolites, exogenous metabolites, metabolites from the gut microbiota and bacterial and host co-metabolites. Metabolic phenotypes revealed significant pattern differences between patients at various CAD stages and those with normal coronary arteries in the current work, suggesting that CAD may involve a universal metabolic disturbance. The metabolites, including PE, PC, PS and sphingolipid metabolites, observed in our study were negatively correlated with AS severity and myocardial markers. The roles of phospholipid metabolites in CVD and metabolic syndrome are contradictory [39, 40]. In recent years, studies have indicated that elevated levels of specific PCs, CM and SMs are characteristic of cardiovascular risks and mortality [41–43], and these substances are abundant in the apical membrane of the gut absorptive epithelium and are considered important for the preservation of structural integrity during exposure to bile salts and enzymes [44]. However, PC-16:0/2:0 was found to be negatively associated with CVD risk factors in population-based study of 990 adolescents [45]. What’s more, a recent research indicated that serum C16:0-CM and SM concentrations are negatively correlated with insulin resistance and metabolic syndrome in Danish individuals [46]. As the lipidomic profile is affected by the complex physiological and environmental factors such as the dietary pattern and medication use, it is difficult to draw the same conclusions from different cohorts. Furthermore, technical aspects such as mass spectrometry conditions may also contribute to the inconsistencies between different studies [47]. Ceramides and sphingomyelin may play a more complex role in the regulation of host AS than previously recognized.

We did not observe the main classes of gut microorganism-dependent metabolites that have been linked to CVD risk, such as TMAO. However, our data showed that the taurine and hypotaurine metabolic module was negatively associated with CAD severity. As a necessary amino acid, taurine could regulate gut micro-ecology, which might be beneficial to health, by inhibiting the growth of harmful bacteria, accelerating the production of SCFA and reducing the LPS concentration [48]. Human clinical studies have reviewed the beneficial effects of taurine in the treatment of hypertension, AS and diabetic cardiomyopathy [49]. In addition, our metabolic profile showed that aromatic compounds such as benzenoids, which are normally generated and biosynthesized by bacterial species, significantly perturbed the development of CAD [50]. Phenolic and indolic compounds are typical products of bacterial metabolism of aromatic amino acids, and dietary phenolic compounds are often transformed prior to absorption. The potential mechanistic participation of these metabolites remains to be further chemically elucidated. Overall, through inter-group comparisons and correlation analysis with clinical indicators, we identified metabotypes that are closely related to the gut microbial metabolism, and these metabotypes exhibited significant alterations with the development of CAD.

Results from epidemiological studies have identified multiple major risk factors responsible for CAD development including hypertension, hyperlipidaemia, insulin resistance, and obesity [51, 52]. Moreover, large-scale studies have revealed that genetic factors can only explain a small part of the variation in disease risk [53]. Recently, studies have provided strong support for the idea that the interplay between microorganisms and the host has a contributory role in atherosclerotic CVD [6–8, 13]. In our research, although we did not find any direct correlation between CAGs and the main CAD phenotype indicator that was mediated by serum metabolites, we were able to further identify the correlation between specific bacteria and different stages of CAD. However, we only conducted cross-sectional study and our data was correlative as well. Moreover, many confounding factors like diet and lifestyle may impair the quality of the associative findings. Long-term follow-up studies and functional studies are urgently needed to reveal the specific bacteria that may contribute to CAD through the production of bioactive metabolites. Nevertheless, tracking individuals from stable atherosclerotic plaques to plaque ruptures and thrombosis is a long process that requires long-range standardized follow-up. Overall, the process of AS progression is considered to be dynamic and complicated, and modulation of the gut microbiota composition may represent a promising diagnostic biomarker or therapeutic target. With an independent validation cohort, our study proved that both CAGs and metabolites may potentially be used together as important markers for CAD subgroup diagnosis.

The gut microbial ecosystem, which is arguably the largest endocrine organ in the body, is capable of producing a wide range of biologically active compounds that may be carried via circulation and distributed to distant sites within the host and thereby influences different essential biological processes of the host [54]. In addition, bacteria in the gut constitute a complex ecosystem in which different species exhibit specialized functions and interact as a community. The bacteria in the human gut may survive, adapt, and decline as CAGs in response to environmental perturbations [55]. Therefore, multi-omic studies may provide an improved global understanding of the functional variations that occur in CAD populations. Further studies are needed to investigate the mechanism of action of the key microbiota and metabolites identified in our study during CAD progression.

## Conclusion

AS is a chronic, long-term pathologic process that is associated with inflammatory reactions. The mechanism responsible for the sudden conversion of a stable situation to an unstable condition is usually plaque disruption, which tends to occur after decades of progression, and these vulnerable plaques may suddenly cause life-threatening coronary thrombosis [56, 57]. Therefore, the identification of an effective and convenient biomarker for monitoring vulnerable plaques is very important for prevention of acute MI. Mounting evidence shows that key members of the gut microbiota might be potential candidates [6, 7, 58], but most studies on the gut microbial variations associated with CAD were limited to case-control studies. Our results showed that alterations in the gut microbial community and serum metabolites in different CAD subgroups and alterations in the gut microbiota were correlated with CAD severity via the mediation of serum metabolites. Furthermore, the combination of specific bacterial CAGs and metabolite modules exhibited potential diagnostic value for differentiating patients with different CAD subtypes. These findings may provide new insights for revealing novel potential aetiologies for AS, understanding the role of gut microbiota in CAD, and modulating gut microbiota as a therapeutic target.

## Materials and methods

### Study design and population

We consecutively recruited 40 healthy volunteers and 161 CAD patients who were hospitalized for coronary angiography in Peking Union Medical College Hospital. Patients who exhibited ≥ 50% stenosis in at least one main coronary artery were diagnosed with CAD. Coronary atherosclerotic burden was evaluated using the Gensini score by two professional cardiologists (Additional file 1: Figure S2a). CAD patients were further divided into three subgroups as follows: (1) SCAD, (2) UA and (3) MI. The detailed diagnose criteria of CAD subgroups are summarized in Additional file 3: Supplementary Methods. For controls, we enrolled subjects who exhibited negative results upon coronary artery CT or coronary angiography examination or were identified as having no CAD-related clinical signs and symptoms. Subjects were excluded if they had gastrointestinal diseases, malignant tumours, autoimmune disorders, infectious diseases, renal dysfunction (severe renal disease creatinine > 3.0 mg/dl), a history of gastrointestinal surgery in the previous year or were administered antibiotics for more than 3 days in the previous 3 months.

All clinical information was collected according to standard procedures (detailed in Additional file 3: Supplementary Methods). For the participants, peripheral venous blood was drawn in the morning the day after admission. Participants were given a stool sampler and provided detailed illustrated instructions for sample collection. Stool samples freshly collected from each participant were immediately transported to the laboratory and frozen at − 80 °C immediately.

In addition, we also included a small verification cohort, which was also divided into control group (*N* = 12), SCAD group (*N* = 11), UA group (*N* = 11) and MI group (*N* = 3), and met the same inclusion and exclusion criteria as the discovery phase cohort. The study was performed in accordance with the principles of the Declaration of Helsinki. Subjects provided written, informed consent for participation in the study.

### Untargeted metabolomics study

Sample analysis was performed on Waters ACQUITY ultra-high-performance liquid chromatography system (Milford, MA) coupled with a Waters Q-TOF Micromass system (Manchester, UK) in both positive and negative ionization modes. In order to detect more metabolites as much as possible, we performed both polar ionic and lipid mode depending on the properties of the serum metabolites. Detailed parameters for the sample preparation and HPLC-MS experiment parameters were provided in the Additional file 3: Supplementary Methods.

The raw data were imported to the Progenesis QI (Waters) for peak alignment to obtain a peak list containing the retention time, m/z, and peak area of each sample [59]. By using retention time and the m/z data pairs as the identifiers for each ion, we obtained ion intensities of each peak and generated a matrix containing arbitrarily assigned peak indices (retention time-m/z pairs), ion intensities (variables) and sample names (observations). The matrix was further reduced by removing peaks with missing values in more than 80% samples and those with isotope ions from each group to obtain consistent variables. The CV (coefficient of variation) of metabolites in the QC samples was set at a threshold of 30%, as a standard in the assessment of repeatability in metabolomics data sets. The nonparametric univariate method (Mann-Whitney-Wilcoxon test) was used to analyse metabolites that differed in abundance between the different subgroups corrected for false discovery rate (FDR) to ensure that the peak of each metabolite was reproducibly detected in the samples. Then, the peak list was imported into SIMCA-P 14.0 software (Umetrics AB, Umeå, Sweden) to acquire clustering information and important variables between the CAD subgroups and the control group. Metabolites selected as biomarker candidates for further statistical analysis were identified on the basis of variable importance in the projection (VIP) threshold of 1 from the tenfold cross-validated OPLS-DA model, which was validated at a univariate level with adjusted *P* < 0.05. The online HMDB database (http://www.hmdb.ca) (version: 4.0) [60] and KEGG database (http://www.genome.jp/kegg/) (updated: September 14, 2016) [61], Lipid maps Structure Database (LMSD) (updated: October, 2017) [62] and METLIN (version: 1.0.5673.40082) [63] were used to align the molecular mass data (m/z) to identify metabolites. The mass error used was 0.005 Da for ms1 and 15 ppm for ms2. MetaboAnalyst (https://www.metaboanalyst.ca) (version 4.0) was used for the identification of metabolic pathways [64].

### Clustering of co-abundant serum metabolites.

Clusters of co-abundant serum metabolites were identified using the R package WGCNA [65]. Signed, weighted metabolite co-abundance correlation networks were calculated for all examined individuals. A scale-free topology criterion was used to choose the soft threshold *β* = 14 for serum metabolites correlations. Clusters were identified with the dynamic hybrid tree-cutting algorithm using a deepSplit of 4 [66]. The serum polar metabolite and serum molecular lipid clusters (labelled P01–P42 and L01–L30, respectively) were collectively termed metabolite clusters.

### DNA extraction and 16S rRNA gene V3-V4 region sequencing

Bacterial DNA was isolated from faecal samples using the bead-beating method as previously described [67]. The extracted DNA from each sample was used as the template to amplify the V3–V4 region of 16S rRNA genes using PCR. PCR amplification, sequencing of the PCR amplicons and quality control of raw data were performed as described previously [68]. A sequencing library of the V3–V4 regions of the 16S rRNA gene was prepared as described previously [69]. The purified products were mixed at an equal ratio for sequencing using an Illumina MiSeq system (Illumina Inc., USA).

### Sequencing data analysis

Operational taxonomic units (OTUs) were delineated at the cutoff of 97% using the USEARCH v.8.0 [70]. The protocol can be found on the website http://drive5.com/usearch/manual/uparse_pipeline.html. The detailed procures were stated in our previous publication [69]. Representative sequences for each OTU were built into a phylogenetic tree by FastTree and subjected to the RDP classifier (RDP database version 11.5, http://rdp.cme.msu.edu/classifier/classifier.jsp) [71] to determine the phylogeny with a bootstrap cut-off of 80%.The sequences of all the samples were downsized to 10,800 (1000 permutations) to match the difference in sequencing depth. α- and β-diversity analyses were performed using Qiime v1.8.0 [72]. Shannon’s index, the observed OTUs, and Chao1 index were evaluated. A normalized OTU abundance table was used for the β-diversity analysis, including principal coordinate analysis (PCoA) based on Bray-Curtis, weighted UniFrac, and unweighted UniFrac distances.

PERMANOVA was used to test for statistical significance between the groups using 9999 permutations. To calculate the variation explained by each of our collected host factors, we performed an Adonis test implemented in R. Each host factor was calculated according to its explanation rate, and *P* values were generated based on 9999 permutations.

### Microbial cluster generation using SparCC

The OTUs shared by at least 20% among all the samples were considered key OTUs. The correlation among 274 key OTUs was calculated by the SparCC algorithm [60] with a bootstrap procedure repeated 100 times, and then correlation matrices were computed from the resampled data matrices. Once the bootstrapped correlation scores have been computed, only OTUs with correlation scores greater than 0.4 were classified into CAGs. Meanwhile, we threshold the *P* value at the desired cut-off < 0.05. The correlation values were converted to a correlation distance (1-correlation value), and the OTUs were clustered using the Ward clustering algorithm via the R package WGCNA. Similar clusters were subsequently merged if the correlation between the CAG’s eigenvectors exceeded 0.8. The CAG network was visualized in Cytoscape (version 3.2.1).

### Spearman multi-omic correlation analysis

Spearman correlations between CAGs, serum metabolite modules and clinical parameters were calculated using R, and both differential abundances of CAGs and CAD-associated metabotypes were tested by the Wilcoxon rank sum test. Wherever mentioned, the Benjamini-Hochberg method was used to control the FDR. The visual presentation of multiple omics correlations was performed using the R. ggplot2 package.

### Feature selection using the random forest model

Using the profiles of CAGs and metabolite modules, the discovery phase samples were randomly divided into a training set and a test set. A random forest classifier was trained on 70% of the samples and tested on the remaining 30% of our samples using the random forest package in R. Then, based on this model, we used another independent cohort for further prediction. We used tenfold cross-validation within the training set. We built an optimal set of variables at the lowest cross-validational error. Thus, the predictive model was constructed using the most important variables, which were further applied for ROC analysis. The performance of the smaller models was measured as AUC when applied to the test set, and the confidence intervals for the ROC curves were calculated using the pROC R package.

## Additional files

Additional file 1: **Figure S1.** Overview of the workflow integrating CAD phenotypes, serum metabolome, gut microbiome. **Figure S2.** Distribution of the Gensini score in each CAD subgroup. **Figure S3.** Orthogonal projection to latent structure-discriminant analysis (OPLS-DA) score plots under polar ionic mode. **Figure S4.** Orthogonal projection to latent structure-discriminant analysis (OPLS-DA) score plots under lipid mode. **Figure S5.** Fine-grained correlation profile of serum metabolite clusters and physiological traits in CAD and control subjects. **Figure S6.** Taxonomic alpha diversity of gut microbiomes among 4 subgroups. **Figure S7.** Clustering of the gut microbiota based on the unweighted UniFrac distances between different groups. **Figure S8.** Spearman correlations between CAGs and major CAD risk factor indicators. (PDF 3080 kb)

Additional file 2: **Table S1.** The extrinsic host factor profile included diet, lifestyle, and stool consistency in CAD and control individuals. **Table S2.** Description of metabolite clusters of serum metabolites under polar ionic mode and their associations with Gensini score, no. of stenosed vessels and cTnI in 201 individuals. **Table S3.** Description of metabolite clusters of serum metabolites under lipid mode and their associations with Gensini score, no. of stenosed vessels and cTnI in 201 individuals. **Table S4.** Composition of the 29 fasting serum metabolite clusters comprising the CAD-positive- and CAD-negative metabotypes in 201 subjects. **Table S5.** Adonis results based on unweighted UniFrac distances. **Table S6.** Multi-omic analysis of the gut microbiome, metabolites and CAD phenotype. **Table S7.** Baseline characteristics of validation-phase subjects. (XLSX 53 kb)

Additional file 3: Supplementary Methods. The supplementary file consist of CAD definitions and phenotype measurements, metadatacollection and statistical analysis method as well as sample preparation details for UPLC-MS. (DOCX 27 kb)

## Abbreviations

ACS: Acute coronary syndrome
AS: Atherosclerosis
AUC: Area under the receiver
BMI: Body mass index
BUN: Blood urea nitrogen
CAD: Coronary artery disease
CAG: Co-abundance group
CK: Creatine kinase
CM: Ceramide
CR: Creatinine
cTnI: Cardiac troponin I
CVD: Cardiovascular disease
ECG: Electrocardiogram
FBG: Fasting blood glucose
GP: Glycerophospholipid
HDL-C: High-density lipoprotein cholesterol
hs-CRP: High-sensitivity C-reactive protein
LC-MS: Liquid chromatography-mass spectrometry
LDL-C: Low-density lipoprotein cholesterol
LPS: Lipopolysaccharide
MI: Myocardial infarction
No. of SV: Number of stenosed vessels
OAD: Oral antidiabetic drugs
OPLS-DA: Orthogonal projection to latent structure-discriminant analysis
OTU: Operational taxonomic unit
PC: Phosphatidylcholine
PCI: Percutaneous transluminal coronary intervention
PE: Phosphatidylethanolamine
PI: Phosphatidylinositol
PS: Phosphatidylserine
PWV: Pulse wave velocity
ROC: Receiver operating characteristic
SBP: Systolic blood pressure
SCAD: Stable coronary artery disease
SCFA: Short-chain fatty acid
SM: Sphingomyelin
TC: Total cholesterol
TG: Triglyceride
TMAO: Trimethylamine-N-oxide
TNF-α: Tumour necrosis factor alpha
UA: Unstable angina
VIP: Variable importance in projection
