# Supplementary material for: Alterations in the gut microbiome and metabolism with coronary artery disease severity
Source: Microbiome. 2019 Apr 26;7:68. doi: 10.1186/s40168-019-0683-9 (PMC6486680; doi:10.1186/s40168-019-0683-9)
Supplement: Supplementary file 1 — Figure S1. Overview of the workflow integrating CAD phenotypes, serum metabolome, gut microbiome. Figure S2. Distribution of the Gensini score in each CAD subgroup. Figure S3. Orthogonal projection to latent structure-discriminant analysis (OPLS-DA) score plots under polar ionic mode. Figure S4. Orthogonal projection to latent structure-discriminant analysis (OPLS-DA) score plots under lipid mode. Figure S5. Fine-grained correlation profile of serum metabolite clusters and physiological traits in CAD and control subjects. Figure S6. Taxonomic alpha diversity of gut microbiomes among 4 subgroups. Figure S7. Clustering of the gut microbiota based on the unweighted UniFrac distances between different groups. Figure S8. Spearman correlations between CAGs and major CAD risk factor indicators. (PDF 3080 kb) [file 40168_2019_683_MOESM1_ESM.pdf]

Additional file 1

Supplementary Figures

Alterations in the gut microbiome and metabolism with coronary artery disease severity

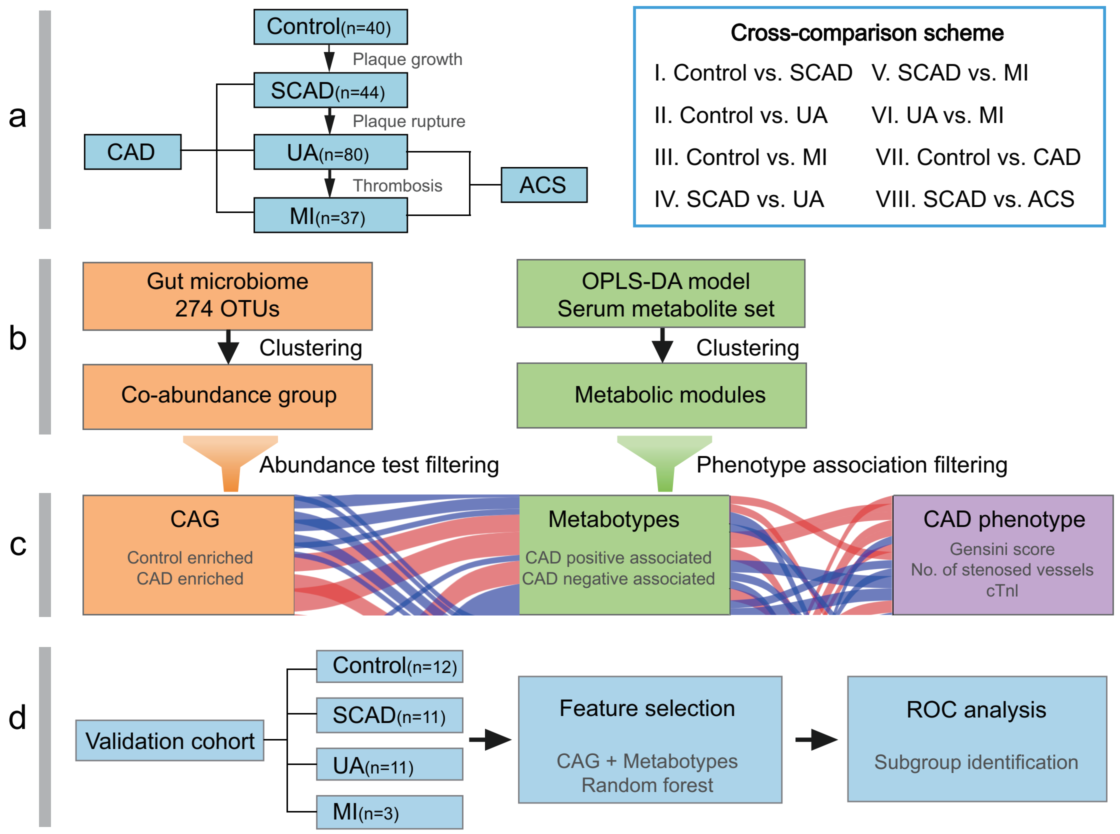

Figure S1 l Overview of the workflow integrating CAD phenotypes, serum metabolome, gut microbiome.

a 161 patients suspected with coronary artery disease (CAD) underwent coronary angiography were divided into appropriate groups: stable coronary artery disease (SCAD), unstable angina (UA), and myocardial infarction (MI) together with 40 healthy controls. **b** We next constructed a co-abundance network with 274 OTUs and clustered them into 24 co-abundance groups (CAGs), and identify the important CAGs that were strikingly different across CAD groups. We then conducted a “cross-comparison scheme” to identify the serum metabolome features in different groups. Serum metabolites were summarized as co-abundance metabolic modules, disturbed features were filtered for significant positive or negative associations with CAD phenotype. **c** Identifying the interrelationship between significantly altered gut microbiota composition, host metabolic profile and main CAD phenotype. **d** Receiver operating characteristic (ROC) analysis of the cross-validated random forest classifier using the most important CAGs and metabotypes in the validation cohort.

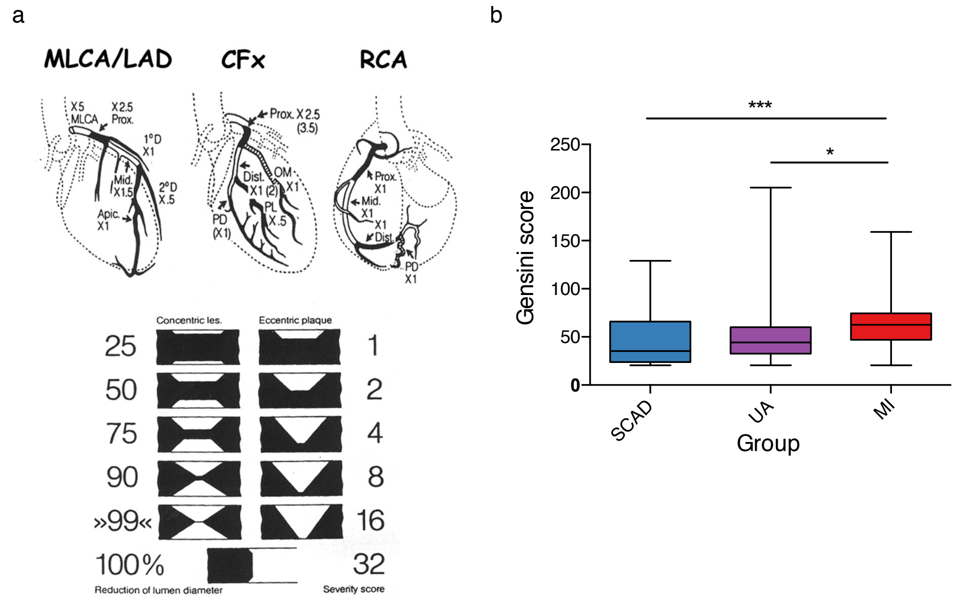

Figure S2 l Distribution of Gensini score in each subgroup of CAD.

a Schematic drawing of Gensini score. The method assigns a different severity score depending on the degree of stenosis, its location (proximal, middle or distal tract) along the target vessel and the type of coronary vessel involved (MLCA, LAD, CFx or RCA). MLCA: main left coronary artery, LAD: left anterior descending, CFx: circumflex, RCA: right coronary artery. **b** Group-level abundance differentiation of Gensini scores. Data are visualized by box-plot. Box represents the interquartile range. The line inside the box represents the median. And whiskers denote the minimum and maximum value. **P* value < 0.05, *** *P* value < 0.001(Kruskal–Wallis test).

Polar ionic mode

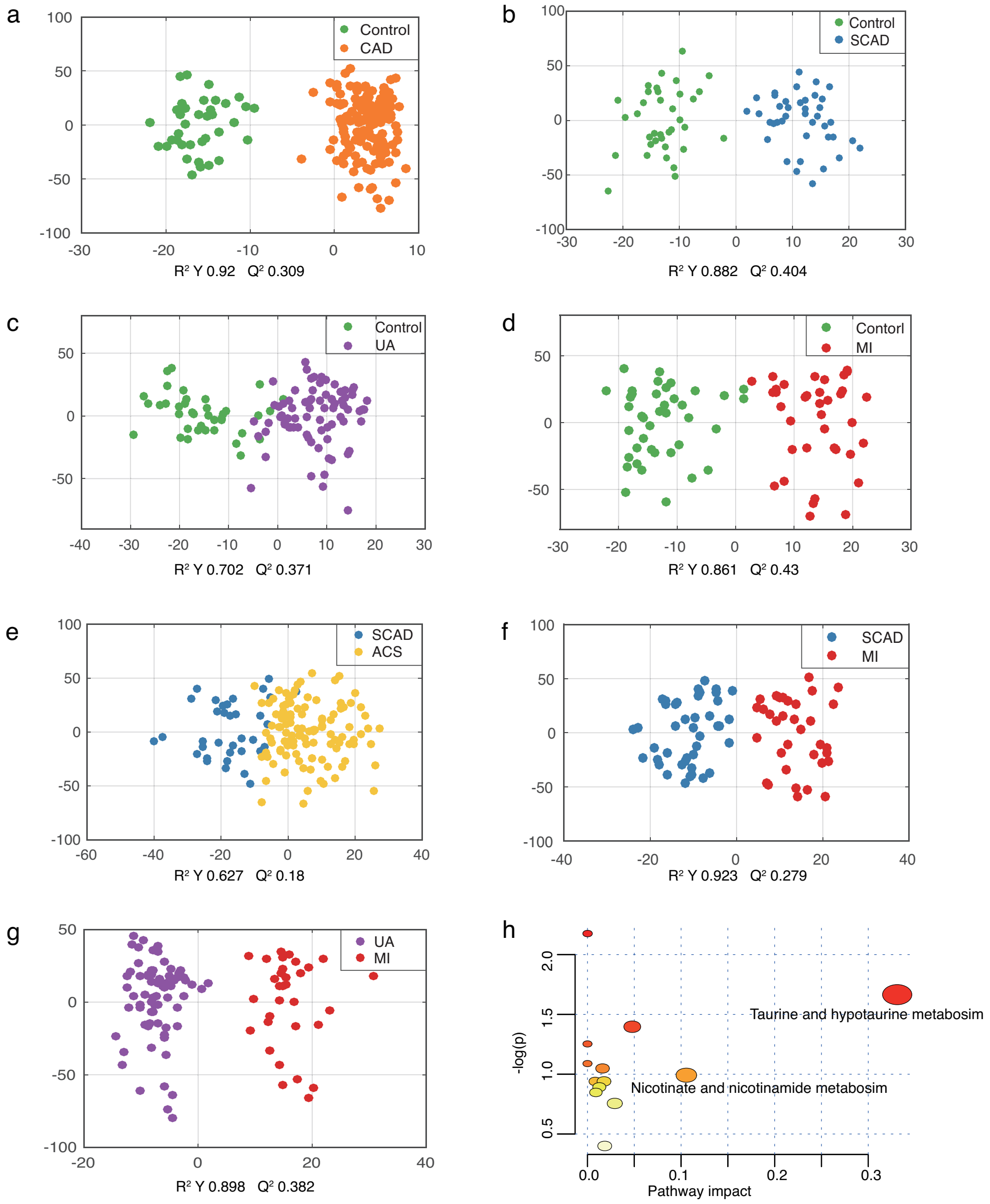

Figure S3 I The orthogonal projection to latent structure-discriminant analysis (OPLS-DA) score plots compared under polar ionic mode.

a Control vs. CAD, **b** Control vs. SCAD, **c** Control vs. UA, **d** Control vs. MI, **e** SCAD vs. ACS, **f** SCAD vs. MI, **g** UA vs. MI, **h** The disturbed metabolic pathways showed various metabolism after combine all significant metabolites into a set.

Lipid mode

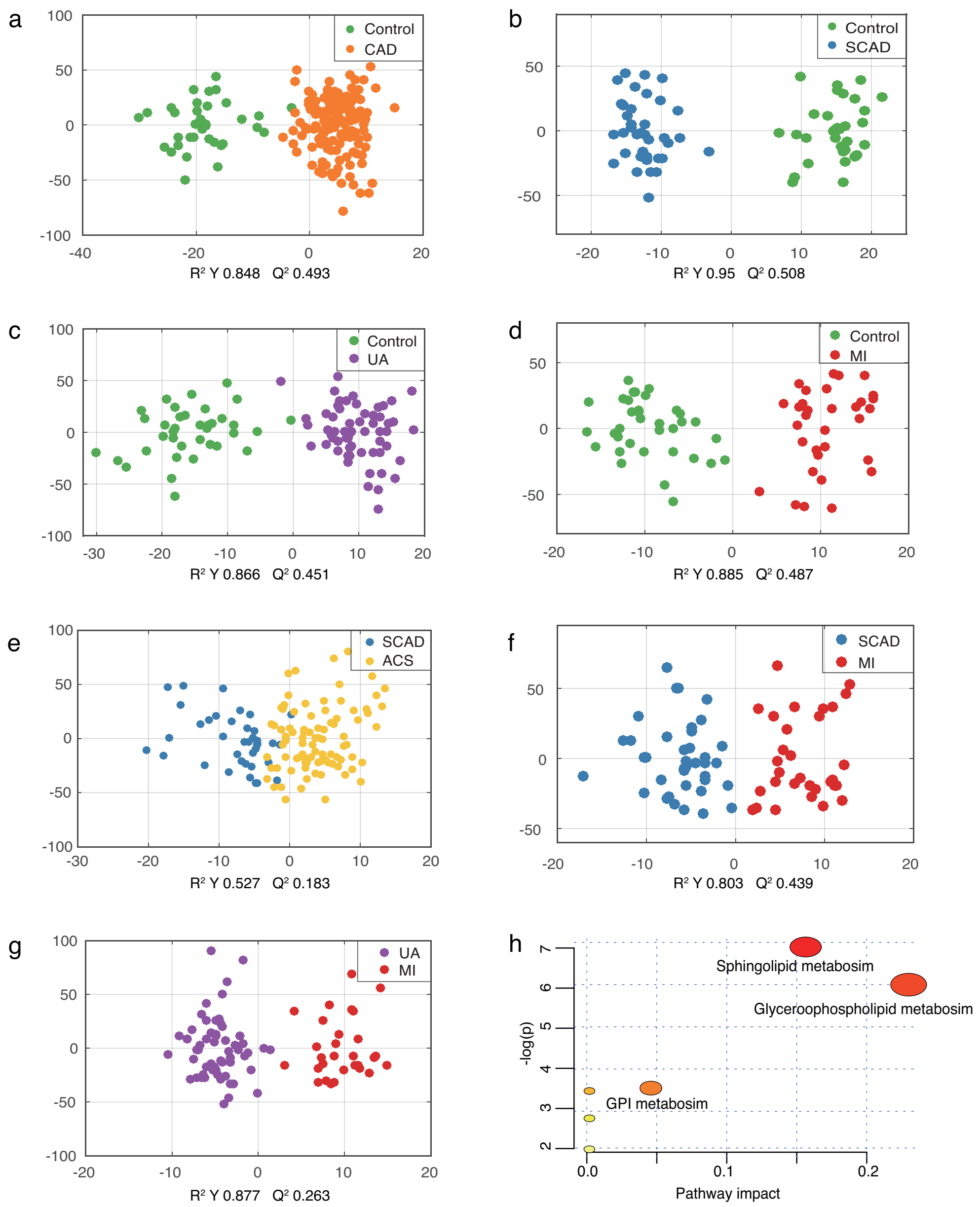

Figure S4 I The orthogonal projection to latent structure-discriminant analysis (OPLS-DA) score plots compared under lipid mode.

a Control vs. CAD, **b** Control vs. SCAD, **c** Control vs. UA, **d** Control vs. MI, **e** SCAD vs. ACS, **f** SCAD vs. MI, **g** UA vs. MI, **h** The disturbed metabolic pathways showed various metabolism after combine all significant metabolites into a set.

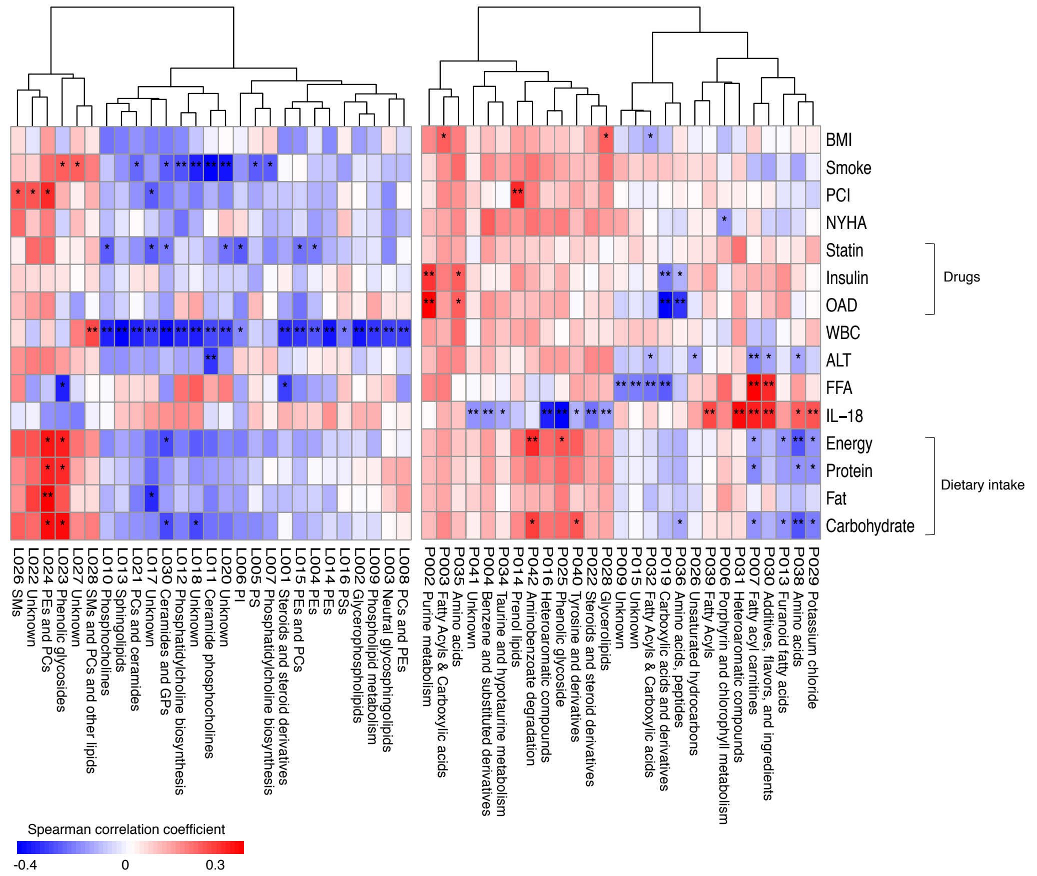

Figure S5 | Fine-grained correlation profile of fasting serum metabolite clusters and physiological traits in CAD and control subjects.

Spearman correlations between all fasting serum metabolite clusters (right panel, serum metabolites under polar ionic mode; left panel, serum metabolites under lipid mode) and clinical phenotypes. The color represents positive (red) or negative (blue) correlations and FDRs are denoted: * , FDR < 0.05; * * , FDR < 0.01. PCI: percutaneous transluminal coronary intervention, NYHA: New York Heart Association (classification), OAD: oral antidiabetic drug, WBC: white blood cell, ALT: alanine aminotransferase, FFA: free fatty acids.

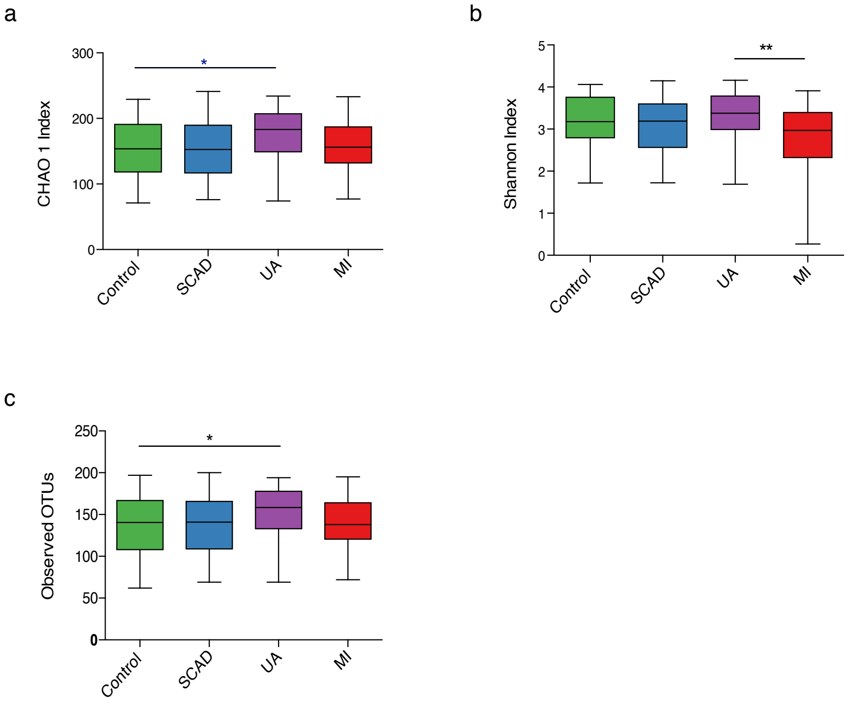

Figure S6 | Taxonomic alpha diversity of gut microbiomes among 4 subgroups.

a Comparison of Chao 1 index between Control, SCAD, UA, MI groups. **b** Comparison of Shannon index between different 4 subgroups. **c** The observed OTUs comparison between different 4 subgroups. Mean, 25th and 75th percentile were shown as boxes. **P* value<0.05, ***P* value<0.01. *P* values are from Kruskal-Wallis test.

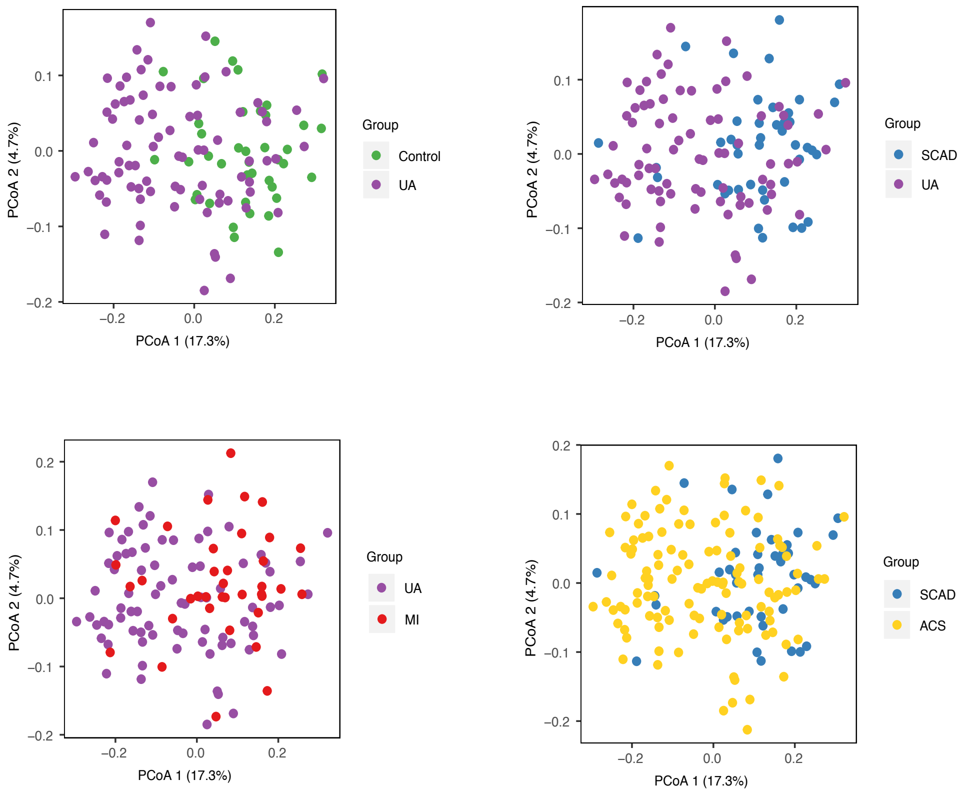

Figure S7I Clustering of gut microbiota based on the unweighted UniFrac distance between different groups.

a PCoA plot based on the unweighted UniFrac distance of gut microbiota samples from Control vs. UA group (*P* value=0.005). **b** SCAD vs. UA group (*P* value=0.0098). **c** UA vs. MI group (*P* value=0.0171). **d** SCAD vs. ACS group (*P* value=0.0384). Differences in beta-diversity were tested by PERMANOVA test, 9999 permutations.

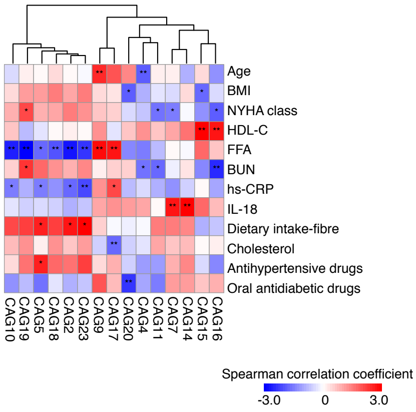

Figure S8 l Spearman correlations between CAGs and major CAD risk factor indicators.

The colour represents positive (red) or negative (blue) correlation, and FDRs are denoted as follows: *, FDR < 0.05; *

*, FDR < 0.01.
