# Supplementary material for: Alterations in the gut microbiome and metabolism with coronary artery disease severity
Source: Microbiome. 2019 Apr 26;7:68. doi: 10.1186/s40168-019-0683-9 (PMC6486680; doi:10.1186/s40168-019-0683-9)
Supplement: Supplementary file 3 — Supplementary Methods. The supplementary file consist of CAD definitions and phenotype measurements, metadatacollection and statistical analysis method as well as sample preparation details for UPLC-MS. (DOCX 27 kb) [file 40168_2019_683_MOESM3_ESM.docx]

**Additional file 3**

**Supplementary Methods**

**Alterations in the gut microbiome and metabolism with coronary artery disease severity**

**Definitions of CAD subgroups**

The diagnosis of SCAD was based on the presence of chest pain that did not change in pattern in the preceding 2 months [1]. UA was defined as a normal measurement of cardiac troponin (cTnI) and with at least one of the following criteria: prolonged (>20 min) angina pain at rest, new onset angina (Class II or III according to the Classification of the Canadian Cardiovascular Society), recent destabilization of previously stable angina with at least Canadian Cardiovascular Society Class III angina characteristics (crescendo angina), or post-myocardial infarction angina [2]. MI was defined as a rise and/or fall of cardiac troponin with at least one value above the 99th percentile upper reference limit and with at least one of the following: (1) ischaemia symptoms; (2) new or presumed new significant ST-segment-T wave changes or new left bundle branch block; (3) development of pathological Q waves in the electrocardiogram; (4) imaging evidence of new viable myocardium loss or new regional wall motion abnormality; and (5) identification of an intracoronary thrombus by angiography or autopsy [3].

**Coronary artery disease phenotype measurements**

Coronary atherosclerotic burden was estimated using the Gensini score (Supplementary Fig. 2) [4]. In brief, concentric lesions and eccentric plaques with 25, 50, 75, 90, 99 and 100% lumen obstruction were angiographically quantified and scored with 1 (for 25% obstruction), 2 (for 50% obstruction), 4 (75% obstruction), 8 (for 90% obstruction), 16 (for 99% obstruction) and 32 (for 100% obstruction) points. Thereafter, a multiplication factor was used depending on the significance of the area supplied by a given coronary segment in which the lesion was present (from 1 to 5). The final coronary artery atherosclerotic score was the sum of all the segment scores (derived from the severity of lumen obstruction multiplied by the factor of segment significance).

**Metadata collection**

The collected metadata covered participants’ anthropometric features and information related to health status, disease history, medication, gastrointestinal conditions, dietary habits, sleep situation, and physical activity. Blood pressure was measured with a standard mercury sphygmomanometer by specially trained nurses when the patient was in a seated position after ≥5 min of rest. Participants' body mass index (BMI) was calculated as weight (kg) divided by squared height (m^2^). Waist circumference was measured by circling the abdomen in a horizontal direction 5 cm above the umbilicus. Drinking history was defined as drinking ≥50 g of alcohol per day.

For the MI patients, peripheral venous blood was drawn from the radial or femoral artery prior to coronary angiography. For the SCAD and UA patients, peripheral fasting blood was drawn in the morning the day after admission. For the controls, peripheral fasting blood was drawn in the morning. Peripheral blood samples were centrifuged at 3000 rpm for 5 min after standing at room temperature for at least 30 min, and the supernatant was purified. Then, the laboratory data were measured immediately.

In face-to-face questionnaire interviews, we collected host metadata, including data regarding gastrointestinal distress, defecation, physical activity intensity, body weight control over one year, dietary intake and Bristol stool score. A meal-based food frequency questionnaire (FFQ) was used to calculate baseline nutrient intake based on [China Food Composition (Book 1, 2nd Edition)]. All information provided during the interviews was recorded in the case report form.

Enzyme-linked immunosorbent assays (ELISAs) were conducted according to the manufacturer's instructions. The ELISA kits for IL-1β, TNF-α, IFN-γ and high-sensitivity IL-6 were all purchased from R&D Systems, Inc. (Minneapolis, MN, USA) and used according to the manufacturer's instructions.

**Statistical analysis of metadata**

Continuous variables were expressed as means with standard deviations in the case of normal distribution or medians with interquartile ranges when variables were not normally distributed. A Student's t-test or, where appropriate, a non-parametric Mann-Whitney U test was used to analyse differences between groups. Categorical data are expressed as numbers and percentages and compared with the chi-squared test or Fisher's exact test. Statistical analysis was performed using IBM SPSS Statistics version 20 (IBM Inc., Armonk, NY, USA).

**Sample preparation for UPLC-MS**

For each precipitation condition, plasma samples (100 μL each) were precipitated by addition of 3 volumes of ACN (polar ionic mode) or IPA (lipid mode) precooled to -20 °C. Samples were vortex mixed for 1 min. After 10 min of incubation at room temperature, samples were stored overnight at -20 °C to improve protein precipitation and then centrifuged at 14 000g for 20 min. The supernatant was collected (600 μL) and stored at -80 °C awaiting MS analysis. The sample was diluted to adjust the water content at 50% and analyzed by UPLC-MS.

**Ultra Performance Liquid Chromatography**

Chromatographic analysis was performed using an Acquity UPLC system (Waters Ltd., Elstree, U.K.). For polar ionic mode, the precipitated and extracted samples (organic phase) were injected onto a HSS T3 column (100 mm × 2.1 mm, 1.7 μm; Waters) at 45 °C [5], and for lipid mode, organic phase were injected onto a C18 CSH column (100 mm × 2.1 mm, 1.7 μm; Waters) at 55 °C [6]. Flow rate was 400 μL/min. Under polar ionic condition, the mobile phase A consists of 0.1% formic acid in H2O and mobile phase B 0.1% formic acid in ACN. While under lipid mode, the mobile phase A consists of ACN/H2O (60:40, v:v) mixed with 10 mM ammonium formate and 0.1% formic acid and mobile phase B IPA/ACN (90:10, v:v) mixed with 10 mM ammonium formate and 0.1% formic acid. The injection volume was 5 μL.

**Profiling by UPLC-Quadrupole-Time-of-Flight (Q-TOF) Mass Spectrometry**

After separation by UPLC, mass spectrometry was performed using a Xevo G2-S Q-TOF for the recovery study with an electrospray ionization (ESI) source (Waters, Manchester, UK) [7]. Dynamic range enhancement was implemented to the mass spectrometry method of the Xevo G2-S Q-TOF in order to improve isotopic distribution and mass accuracy and reduce high ion intensities. In positive ion-mode, MS parameters were as follows: capillary voltage was set at 2.5 kV, cone voltage at 30 V, source temperature at 120 °C, desolvation temperature at 400 °C, desolvation gas flow at 800 L/h, and cone gas flow at 20 L/h. Acquisition was performed from m/z 50 to 1200 under polar ionic condition and 100 to 1500 under lipid condition. In negative ion mode, MS parameters were as follows: capillary voltage was set at 2.5 kV, cone voltage at 25 V, source temperature at 120°C, desolvation temperature at 500 °C, desolvation gas flow at 800 L/h, cone gas flow at 25 L/h. The scan range was as described above. For both ionization modes, leucine enkephalin (m/z 556.2771 in ESI+, m/z 554.2615 in ESI-) was continuously infused at 30 μL/min and used as lock mass correction.

**References**

1. Montalescot, G., et al., *2013 ESC guidelines on the management of stable coronary artery disease: the Task Force on the management of stable coronary artery disease of the European Society of Cardiology.* Eur Heart J, 2013. **34**(38): p. 2949-3003.

2. Hamm, C.W., et al., *[ESC guidelines for the management of acute coronary syndromes in patients presenting without persistent ST-segment elevation. The Task Force for the management of acute coronary syndromes (ACS) in patients presenting without persistent ST-segment elevation of the European Society of Cardiology (ESC)].* G Ital Cardiol (Rome), 2012. **13**(3): p. 171-228.

3. Thygesen, K., et al., *Third universal definition of myocardial infarction.* Glob Heart, 2012. **7**(4): p. 275-95.

4. Gensini, G.G., *A more meaningful scoring system for determining the severity of coronary heart disease.* Am J Cardiol, 1983. **51**(3): p. 606.

5. Dunn, W.B., et al., *Procedures for large-scale metabolic profiling of serum and plasma using gas chromatography and liquid chromatography coupled to mass spectrometry.* Nat Protoc, 2011. **6**(7): p. 1060-83.

6. Sarafian, M.H., et al., *Objective set of criteria for optimization of sample preparation procedures for ultra-high throughput untargeted blood plasma lipid profiling by ultra performance liquid chromatography-mass spectrometry.* Anal Chem, 2014. **86**(12): p. 5766-74.

7. Zelena, E., et al., *Development of a robust and repeatable UPLC-MS method for the long-term metabolomic study of human serum.* Anal Chem, 2009. **81**(4): p. 1357-64.
